# Relationship between Pituitary Gland and Stem Cell in the Aspect of Hormone Production and Disease Prevention: A Narrative Review

**DOI:** 10.2174/0118715303314551241031093717

**Published:** 2025-01-13

**Authors:** Amit Sharma, Rohit Kumar, Arti Saini, Wandeep Dagar, Kanishka Kapoor, Karan Goel, Isha Chawla, Meenakshi Dhanawat

**Affiliations:** 1 Department of Pharmacy Practice, MM College of Pharmacy, Maharishi Markandeshwar (Deemed to be University), Mullana - 133207, Ambala, Haryana, India;; 2 Department of Pharmacology, MM College of Pharmacy, Maharishi Markandeshwar (Deemed to be University), Mullana - 133207, Ambala, Haryana, India;; 3 Amity Institute of Pharmacy, Amity University Haryana, Gurugram, Haryana -122413, India

**Keywords:** Anterior pituitary, neural ectoderm, adenohypophysis, rathke’s pouch, organizing center, stem cell

## Abstract

**Objectives:**

In the last two decades, scientists have gained a better understanding of several aspects of pituitary development. The signaling pathways that govern pituitary morphology and development have been identified, and the compensatory relationships among them are now known.

**Aims:**

This paper aims to emphasize the wide variety of relationships between Pituitary Gland and Stem cells in hormone Production and disease prevention.

**Methods:**

Based on many case reports and several types of research, a wide variety of relationships between the Pituitary Gland and Stem cells in the aspect of hormone Production and disease prevention are reviewed in this literature. In this paper, we focus on various roles and functions of the pituitary gland, the responsibilities of stem cells as a mode of hormone production, and disease prevention.

**Conclusion:**

Within this period, more was discovered concerning the contributions made to the transcription factors within the pituitary development, with factors such as Prop1, Pitx1, and Sox2 being defined as important in the development and action of hormone-secreting cells. They are also required in the appropriate specification of the cell types in the pituitary gland and the persistence of the progenitors. Manipulation of these factors causes developmental defects as well as tumors, thus the necessity of knowing the precise function and interaction of these factors. A closer look at these transcription factors could help expand treatment options for structural defect development or give rise to pituitary adenomas.

It has been established that signaling pathways such as Sonic Hedgehog (Shh), Wnt, and Notch play a part in modulating pituitary development. These pathways are involved in regulating important processes such as cellular proliferation, differentiation, and organization of the pituitary gland tissues. Breaching any of these pathways has been correlated with the development of various pituitary-related conditions including adenomas and congenital hypopituitarism. Moving forward, further studies of these pathways and their associations with stem cells could provide a better understanding of disease processes and approaches to manage them. This way, there is a possibility of developing new approaches aimed at treating the cause of the dysfunction of the pituitary gland by modulating its specific signaling activities.

Promising directions for the stimulation of hormone synthesis and restoration of normal pituitary function upon its disorders *via* tissue regrowth could be found in stem cell application. The fact that one can generate functional pituitary cells from iPSCs for instance provides new avenues both for the understanding of pituitary disease mechanisms as well as for personalized medicine. It is possible to utilize these stem cell-derived cells for modeling disease, drug discovery or even transplantation to restore the function of the damaged pituitary gland. In the future, however, the focus ought to be on the effective application of stem cell therapies that have been research during the development of better differentiation processes.

The recent understanding of the system that carries the hypothalamic hormones to the pituitary gland, *i.e.,* the hypophyseal portal vasculature, has had its implications too. This factorial consideration emphasizes the role of the vascular component in the control of pituitary activity – the release of hormones by the pituitary gland. Exploring stem cell-hypophyseal portal system interactions may open new avenues of treatment for diseases associated with deficient hormone transportation and/or pituitary dysfunction.

## 1 INTRODUCTION

Due to its ability to control metabolism, growth, reproduction, and stress response, the pituitary gland is frequently referred to as the “master gland” of the body. Prolactin (PRL), follicle-stimulating hormone (FSH), luteinizing hormone, gonadotropins, growth hormone (GH), adrenocorticotropic hormone (ACTH), thyroid stimulating hormone (TSH), lactotrophs, somatotrophs, thyrotrophin, corticotrophs, and gonadotrophs (LH) are a few of the specialized cell types in the anterior pituitary gland that secrete different hormones. The physical stages of the pituitary gland's development in many species have been documented since at least a century ago [1, 2]. The current understanding of the growth, development, and disease condition of the pituitary gland has been affected by lessons acquired from fish (zebrafish), amphibians (bullfrogs), mammals (mouse and rat), birds (chick-quail), and amphibians. Several investigations developed the framework for the signaling that governs the regulation [3-6]. A magnified microscope made it possible to identify different types of hormone-producing cells based on their physical appearance. Later, antibodies specific to individual hormones made it possible to track the emergency response of separating cells during the stage of embryogenesis [7-9]. Signaling molecules found in early transplant experiments and hallmark transcription factors required for cell selection and lineage determination were discovered [10-12].

**Fig. (1):**
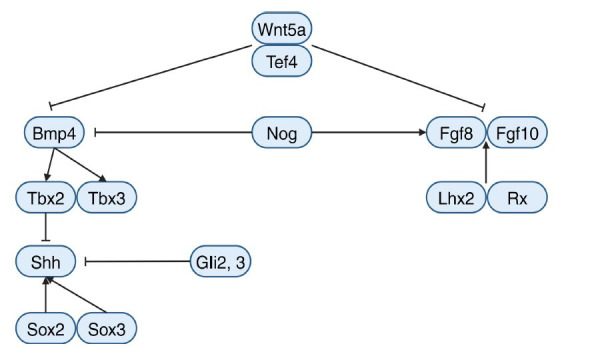
The anterior pituitary gland's growth and shape are regulated by signaling pathways that start in the organizing center.

The biology of stem-like frameworks in the embryo and adult pituitary gland progenitors is currently the subject of a lot of research [13, 14]. Progenitor recruitment, pregnancy-related hypertrophy, puberty, and excessive external stresses are among the less-studied subjects. A more profound comprehension of pituitary development begins with understanding the development of the circulation to the hypothalamus, which is essential for the pituitary gland to receive hormones from the brain and to distribute hormones to target organs [15]. Researchers are looking at how pituitary cell networks evolve and how they contribute to the production of hormones [16]. Many familial pituitary adenomas and congenital pituitary hormone deficits have been reduced due to research on pituitary development (Fig. **1**).

## 2 CONTROLLING THE SIZE AND STRUCTURE OF RATHKE'S POUCH AS WELL AS THE PITUITARY ORGANIZER

The pituitary gland comprises two ectodermal components - the neural and surface ectoderm. The rear lobe is created by the neural ectoderm, while Rathke's pouch, the progenitor of the front and middle lobes, is produced by the surface ectoderm. In this context, the formation and patterning of the rear lobe (or neurohypophysis), which originates from the ventral diencephalon, are examined in detail. The ventral diencephalon holds a key role in the growth of the pituitary gland by forming the posterior lobe and setting the gland's dimensions and structure. The ventral diencephalon's organizing core is made up of overlapping expression patches of fibroblast growth factors and bone morphogenetic protein, which together form the infundibulum [17-21]. The infundibulum and the organizing center are located rostral to a region of sonic hedgehog (SHH) expression [21]. Rathke's pouch creation requires BMP4 as an inductive signal as Bmp4-/-mice does not create the pituitary placode or Rathke's pouch [5]. After Rathke's pouch is created, cell growth within it requires FGF signaling. The pouch is created when Fgf10-/- and Fgfr2IIIb-/-mutants are present, but it is unable to proliferate and is ultimately destroyed by apoptosis [22, 23]. The ventral diencephalon of mice deficient in the Nkx2.1 gene expresses less Fgf8, which causes a hypoplastic Rathke's pouch. Mice lacking the Fgf10 gene in their pituitary glands exhibit behavior akin to this [5]. Mice with hypomorphic mutations in Fgf8 exhibit several distinct phenotypes, including an enlarged anterior pituitary lobe, a missing posterior pituitary lobe, and anomalies in the neural ectoderm midline, such as holoprosencephaly [24]. During the initial stages of pouch formation and growth, BMP and FGF have significant roles, and their dosage sensitivity has been confirmed. The pituitary gland expresses of many FGF family genes, and the precise roles of FGF8 and FGF10 in the pituitary coordinating unit are yet unknown. While FGF10-/- mice show no defects in the midline, FGF8 is essential for developing the neuroectoderm midline [23-25]. Like FGF3 and FGF10's interdependence in building the chick infundibulum, FGF8, and FGF10 may also cooperate in the development of the mouse infundibulum. Researchers should look at Fgf10 expression in Nkx2.1-/- and Fgf8 hypomorphic mutants as well as Fgf8 expression in Fgf10-/- animals to comprehend the compensatory changes in expression. It is noteworthy that FGFs 13, 14, and 17 are included in the embryonic pituitary transcriptome [26-28], whereas the organizing center expresses FGF18 [26, 27]. The FGF family has functional redundancy. A gene defect in one signaling pathway can change gene expression in another pathway. BMP4 activity is inhibited by noggin. In Nog-/-mice, a greater domain of BMP4 and a lower level of FGF10 suggest that communication between the two pathways in the ventral diencephalon is occurring [29]. The pituitary gland displays diverse dysmorphologies triggered by a larger surface area of the ectoderm during development. Wnt signaling affects the production of FGF and BMP. Mutations in Wnt5a cause minor dysmorphology, whereas Tcf7l2 mutations substantially increase pituitary gland size [30-33]. WNT5A acts *via* a non-canonical Wnt signaling pathway, resulting in less stable beta-catenin in the nucleus. A lack of transcriptional regulation causes tcf7l2 mutants' overgrowth. Wnt11 and Wnt16 activate non-standard and standard WNT signaling pathways, respectively, in the ventral diencephalon during pituitary development [33]. Through TCF7L2, Wnt16 can regulate the pituitary organizer. Non-canonical WNT5A signaling is mediated by ROR1 and ROR2 receptors. to Wnt5a-/- animals, Ror1-/- and Ror2-/-mice have limb truncation, but they do not have the pituitary phenotype [34]. Further research is required to identify the crucial components and recognize the functions of classical and non-classical WNT signals in the formation of pituitary organizers.

The pituitary gland and its surrounding tissues develop while containing different members of the WNT gene family. There are two types of WNTs detected in the ventral diencephalon: Wnt11 and Wnt16. Wnt11 is a non-canonical WNT, while Wnt16 is a canonical WNT [33]. Wnt16 is a promising option for regulating the pituitary organizer. ROR1 and ROR2 receptors mediate the non-canonical response to WNT5A signaling. Further research is necessary to determine the key factors and roles of WNT signaling in pituitary organizer formation [34].

SHH has a role in pituitary growth and is controlled by transcription factors. The pituitary organizer and oral ectoderm of the ventral diencephalon express Sonic Hedgehog. The pituitary placode originates from an oral ectoderm region that is SHH-negative. In the ventral diencephalon, SHH expression limits the development of the pituitary gland. Pituitary hypertrophy results from the ventral diencephalon's loss of Shh function [35]. The Shh enhancer SBE2 is bound by SOX2 and SOX3 accumulation, which then promotes expression and regulates Shh transcription in the ventral diencephalon. The pituitary organizer expands in response to a dose-dependent deletion in Sox2 and Sox3, which is brought on by lower Shh expression in the ventral diencephalon [35]. SOX2 and SOX3 are inhibited from functioning by T-box transcription factors (TBX2 and TBX3). Tbx3 proteins bind to specific DNA sequences (SOX2 and SOX3) and avoid activating Shh expression through the SBE2 enhancer. In Tbx3 deficient animals, there is an uplifting SHH expression and a downfall in BMP4 and FGF10 expression, leading to pituitary gland under-development [36]. The Gli transcription factors Gli2 and Gli3 are crucial in SHH signal transmission. GLI2 is mainly responsible for activating SHH transcriptional targets, while GLI3 primarily suppresses them [37]. In Gli2-/- embryos, the pituitary organizer and hypomorphic pituitaries exhibit reduced Bmp4 and Fgf8 expression levels. Conversely, in Gli2-/- and Gli3-/- embryos, the pituitary gland is completely absent [38]. Regulatory mechanisms of the pituitary-organizing center are complex, involving GLI proteins with active and repressive roles. It is unknown if GLI2 and GLI3's repressive action is required to guarantee the expression of Bmp4 and Fgf8, or if early and vigorous SHH signaling is required to induce these expressions in the pituitary organizer [39]. LHX2 and RX, which are homeobox transcription factors containing LIM and paired-type homeodomains, respectively, regulate the pituitary-organizing center. Due to mutations in these factors, the ventral diencephalon expresses more BMP4 and FGF8 but less SHH, resulting in bigger pituitaries. While FGF8 and BMP4 levels are predicted to increase, FGF10 expression is decreased in Rx-/- mice [40]. These mouse models may provide further insights into the transcriptional modulation of the pituitary activator and Rathke's pouch induction.

### 2.1 Significance of Response Signaling Pathway Activities Specific to Rathke's Pouch

Rathke's pouch and surrounding areas include BMPs and FGFs, both of which have unclear roles in the specification of anterior lobe cells. The gain of function models suggests that excessive signaling may impact cell distinction and the size of cell-cell populations. Functional response models shed light on how excessive signaling affects the number of cells given in each population as well as how quickly they proliferate. Conversely, loss of function models highlights how signaling response molecules help shape and develop cells, but do not alter their structural makeup. FGF8 and FGF10 are expressed in the infundibulum, dorsal to Rathke's pouch, during mouse development at e10.5, whereas BMP is documented on the ventral side and surrounding mesoderm [20, 21]. Interactions between BMP and FGF signaling influence anterior lobe cell differentiation. Progenitor cells closer to somatotropes become somatotropes, while those closer to BMP2 become gonadotropes. No studies have yet traced progenitor cells from a specific starting point to a predetermined endpoint. A cell's final location in the anterior lobe may not directly relate to its position in Rathke's pouch [41]. A study on birth dates has found that progenitor cells, which cease cell division at the same time, are present throughout the front lobe. This suggests that there is an active migration of cells. However, it is unclear if progenitor cells close to the lumen have identical characteristics or if their attributes vary based on their position in Rathke's pouch before cell division ends. As they finish cell division, the cell types in the anterior lobe begin to divide between e11.5 and e13.5 [42, 43]. Progenitor cells express p57Kip2 (Cdkn1c), indicating that they have transitioned from cycling to an undifferentiated state after exiting the cell cycle [44]. As they leave the lumenal epithelial and go closer to the anterior lobe, these cells may be seen on the underside of the lumen. At e11.5, which is also the start of cell cycle departure, Rathke's pouch explants develop resistance to exogenous signals like FGF and BMP [20, 21]. This indicates that signals from Rathke's pouch are likely to encourage cell growth. Changes in BMP and FGF levels in the pituitary organizer do not impact the development of cells in the anterior lobe [29, 31, 33, 39].

To fully understand the functions of WNT and BMP in Rathke's pouch, more investigation is essential. There may be a purpose for the pouch's expression of BMP2, WNT4, WNT6, WNT11, and WNT16 [21, 29, 33]. Expression of Rathke's pouch of a dominant negative Bmpr2 receptor significantly reduces BMP signaling, leading to the loss of corticotrope development and the POU1F1 lineage, which comprises somatotropes, thyrotropes and lactotrophs [21]. In Rathke's pouch, BMP4 expression stimulates BMP signaling, which leads to an increase in intermediate cell differentiation, particularly in cells expressing Gata2 and Isl1. However, it prevents the terminal development of hormones except corticotropes [21]. Determining whether the non-physiological gain of function test findings adequately reflects the intrinsic functions of signaling networks is challenging. Expression of many WNTs in Rathke's pouch indicates the use of both classical and non-classical pathways and functional redundancy—wnt4 deficiency results in decreased somatotropes and thyrotropes [21, 33]. Hesx1-cre alters cell specification, reducing all cell types except corticotropes and increasing pituitary stem cells, which results in craniopharyngiomas [45, 46]. Nucleus localized catenin cannot differentiate outside the stem cell niche, even though all anterior lobe cells contain a gene. This is what causes the protein to produce a version that is resistant to destruction. It appears that the anterior pituitary contains mechanisms to prevent β-catenin from being activated [6]. The observed diverse phenotypes are most likely the result of these two cre drivers expressing differently throughout time and space. Moreover, cre expression has a distinct starting role [47-49] when it is acquired at the cost of an endogenous allele. Despite originally being expressed across the oral ectoderm, SHH is not found in Rathke's pouch placode. Nevertheless, it appears that Rathke's pouch cells are producing SHH signals due to the expression of the downstream target gene patched (Ptc1) [50]. Overproduction of SHH increases Bmp2 expression in Rathke's pouch, along with thyrotropes and gonadotropes [50]. Progenitor proliferation is reduced in embryos with conditional Gli2 inactivation in the pituitary. Still, the pituitary is formed correctly, and the hormone-producing cells are unaffected other than a decrease in corticotropes [38]. The gain of function may outweigh the capacity for adaptability in loss of function mutants. Ectopic synthesis of SmoM2, which promotes proliferation without altering cell specification, activates Shh signaling in the pituitary [38]. The distinctions between Gli2 and HIP transgenic studies may point to a broader mechanism of HIP, like blocking Rathke's pouch and ventral diencephalon SHH signaling, or non-canonical SHH signaling in the pituitary, like non-Gli-dependent activation of RAC1 or RHO [51]. Since Ptc1 plays roles that are independent of Smo and Gli, non-canonical SHH signaling may account for the variations in outcomes seen when the SHH pathway is stimulated ectopically in Rathke's pouch [51]. According to recent research, the Notch signaling system has a role in anterior lobe cell determination as well. Because of the Notch-responsive transcription factor, Hes1-deficient embryos exhibit a cell fate transition from melanotropes to somatotropes [52]. POU1F1 lineage and corticotrope differentiation are lost by conditional ablation of Rbpjk, an intracellular regulator of Notch signaling, utilizing Rathke's pouch [53-57]. To summarize, WNT, Notch, FGF, and BMP are essential for the proper functioning of the pituitary gland and its surroundings. Due to the increased complexity, comprehending the intricate web of interactions among pathways, ligands, and receptors poses a significant challenge. Further research is required to gain a complete understanding of the roles that various signaling pathways play in cell specification and the potential compensatory adjustments that may be implemented to ensure the appropriate distribution of cell types in the pituitary anterior lobe.

### 2.2 Signature Response Transcription Factors and Their Function in Cell Initiation

**Fig. (2):**
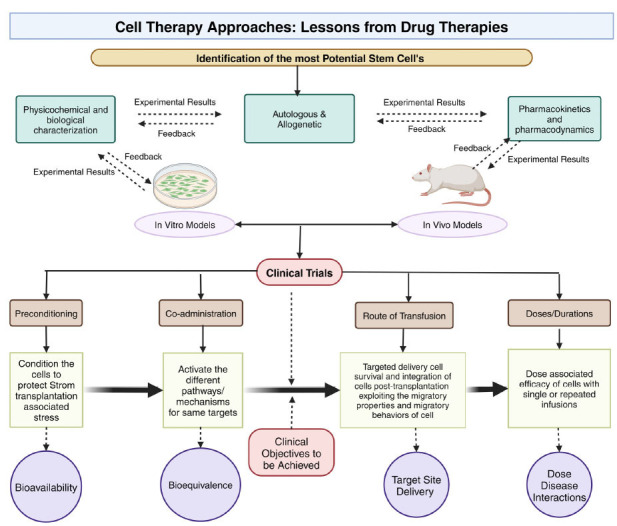
Lessons from drug therapies for cell therapy approaches.

Numerous transcription factors are important in human disease, and they play crucial roles in the specification. For instance, POU1F1 activated the prolactin and growth hormone genes, which led to its discovery [58-62]. Mutants lacking POU1F1 are unable to produce these cells. Using similar techniques, several critical transcription factors were identified [63-67]. In other cases, partial differentiation rather than the complete absence of the cell type results from transcription factor deficiency. For instance, mice lacking NR5A1 are unable to produce gonadotropins; nonetheless, gonadotrope differentiation may be effectively induced by hyperstimulation with GnRH, indicating that NR5A1 is not necessary for gonadotrope differentiation [68]. Similarly, although neither NeuroD1 nor TPIT are essential for corticotrope formation, their absence delays or reduces POMC expression [65, 66]. Neglecting to promote deviation from a specific path might lead to alternative options. Since POU1F1-dependent somatotropes rather than melanotropes are present in the hypoplastic middle lobe, HES1 deficiency may potentially be the cause of ectopic differentiation in the intermediate lobe [67-76] (Fig. **2**).

The present state of the art needs to acknowledge the mechanisms causing this illness to arise as well as the epigenetic regulation that permits transcription factors to bind to chromatin. Deconstructing the differentiation processes of hormone-producing cells using genome-wide modification and evaluation of transcription factor binding sites and DNase-sensitive open chromatin is a powerful method. One recent example is PAX7, a new transcription factor that plays a crucial role in regulating enhancers from several genes, either by blocking binding or expanding the chromatin to facilitate TPIT binding [77].

Lacking the PAX7 selector, middle columnar lobular cells separately form corticotropes instead of melanotropes. This endeavor's primary focus will be comprehending comparable origins and selecting mechanisms for many cell types that produce hormones. These studies are incredibly challenging due to the intricacies of the separation process in embryonic pituitary tissue, which is rare and difficult to replicate with current cell processing techniques. (Table **1**).

### 2.3 Transcription Factors That Operate in Early Phase Transmission

PROP1 is the 1st identified transcription factor. Numerous genes, each crucial to the organogenesis process, depend on this paired homeodomain protein to be activated or silenced. It is necessary for the timely silencing of HESX1 and OTX2 and the initial activation of POU1F1 and NOTCH2 [55, 78-81]. Although some evidence indicates that beta-catenin must be significantly suppressed for healthy development, beta-catenin may govern PROP1's switch from repressor to activator [31, 45, 46]. In humans, PROP1 deficiency affects hormone-generating cell types [82], whereas in mice, gonadotropin, PRL, TSH, and GH are congenitally absent [83, 84]. PROP1 absence and proliferating cell associations in Rathke's cleft simulate a failed epithelial-to-mesenchymal transition by failing to delaminate [85, 86]. In mice, this results in a very hypoplastic and dysmorphic organ, while in humans, it creates a broad variety of organ morphologies [76, 85]. Several kinds of pituitary cells are targets of transcription factors that act prematurely in pituitary growth. In contrast to PROP1, most of these genes are not limited to the pituitary gland; rather, their mutations affect other developing organs, potentially resulting in syndromic hypopituitarism [76]. Humans are probably fatally affected by the loss of functional changes in several genes due to their pleiotropic effects. PITX2, PITX1, LHX3, LHX2, LHX4, and OTX1, OTX2 have varied craniofacial and pituitary characteristics, probably due to functional alteration in the belonging members of the similar gene family. Many of these genes have mutations that cause decreased proliferation and increased apoptosis [49, 87-89].

In the developing mouse embryo's skull, OTX1, OTX2, EMX1, and EMX2 show overlapping expression patterns [90]. Gene targeting tests revealed that each of these transcription factors is essential to embryogenesis and that the genes in the gene family balance one another out. At the same time, Otx2+/- heterozygotes exhibit a wide range of craniofacial abnormalities, from pituitary hormone problems (aplasia) to severe pituitary hypoplasia and dysmorphology, Otx2-/- animals lack head structures anterior to rhombomere 3 [91]. Mutants lacking Otx1 exhibit a less severe phenotype, characterized by a brief postponement of growth development and puberty [92]. This suggests that Otx1 and Otx2 are involved in the pituitary gland's growth and development process, with Otx2 being more crucial to maintaining balance in organ formation and functional processes. Otx2 is highly expressed in the neural ectoderm, which generates FGF and promotes the development of Rathke's pouch [79]. When Pou1f1 transcription begins, Otx2 expression in Rathke's pouch is modest and fleeting, with a restricted appearance observable.

Given that the growth of the pituitary stem and hind lobe depends on OTX2, it follows that OTX2 mutations cause hypopituitarism in both humans and mice. It is thought that a neural ectoderm shortage, which results in fewer inductive impulses coming from the organizing center, is the cause of anterior lobe hypoplasia. Benefits from functional stacking extend beyond those sharing a common gene prototype. Other genes, which vary throughout inbred strains, can either cause or prevent the Otx2 mutant phenotype in addition to the EMX and OTX genes [91]. Otx2 heterozygotes with a C57BL/6 background are more prone to severe craniofacial abnormalities, whereas CBA protects them from these consequences [93, 94]. On a mixed background of B6 and CBA, otx2 heterozygotes exhibit mild to severe acephalic [95-97].

## 3 NEW FUNCTIONS FOR OTHER GROUPS OF TRANSCRIPTION FACTORS: THE FORKHEADS

Transcriptome research has revealed that the pituitary gland has unique genes whose functions are unknown [28]. Pituitary development has been associated with the SIX gene family [98, 99]. Common effects of the group on pituitary and ocular growth provide credence to the theory those tissues share regulatory circuits. Brinkmeier and Davis's publications state that many HMG boxes, homeobox, orphan nuclear receptors, and helix-loop-helix still need investigation. Examining these novel genes' expression during development is the first step towards comprehending their purpose. As the role of forkhead genes in pituitary development becomes more apparent, it illustrates the difficulty that may characterize other, as-yet-undiscovered gene groups. Forkhead transcription factors are named after a given origin to a similar category. They contain a reserved, double helix DNA. Forkheads have been connected to several physiological processes, such as chromatin remodeling, metabolism, and development [100, 101]. There are fifty known biomarkers in humans and forty-four in mice. Standardized applications have been applied to forkhead marking factors. Forkhead Box is a subfamily identified by a number system [102, 103]. Forkhead gene mutations commonly result in autosomal dominant disorders with haploinsufficiency in humans.

The first pituitary forkhead identified was FOXL2, often called Pfrk [21]. FOXL2 participates in female sex determination [100, 106, 107] and ovarian growth and function [104, 105]. A mutated human with the FOXL2 gene causes Ptosis, Blepharophimosis, and Epicanthus Inversus Syndrome (BEPS), a loss of function disease that results in deformed eyelids and premature ovarian failure [106-108]. Although homozygous mutant mice demonstrate that FOXL2 has a role in pituitary regulation, describing that both Foxl2 alleles must be deleted to impact pituitary regulation, humans with BPES do not have pituitary abnormalities [109-112]. Human and mouse gonadotropes and thyrotropes have been shown to express FOXL2 [113, 114]. FOXL2 is involved in gonadotrope differentiation and proliferation since it appears in most null cells and gonadotropin smaller unit-making adenomas [115]. Clusterin and FOXL2 work together to regulate the growth of gonadotroph adenoma. Together with SMAD3, FOXL2 regulates the follistatin (Fst) activin responsiveness in T3-1 cells [116, 117]. It also activates the Gnrhr gene promoter's activin-responsive region. However, Gnrhr expression does not need FOXL2, suggesting that there could be genetic [109]. In transgenic mice, the state of FOXL2 ectopic expression is sufficiently rigid to cause ectopic expression of the Cga gene, which codes for the glycoprotein hormone subunit (GSU) [113]. Since Cga expression is decreased in Foxl2 knockout mice while transcripts in a pituitary-specific deletion of FOXL2 are normal, it is unclear if FOXL2 is necessary for Cga expression [109, 118].The hypothalamic contributions of FOXL2 to the regulation of Cga expression or the timing or effectiveness of Foxl2 deletion in animals exhibiting conditional knockouts might perhaps account for this apparent disparity. The gene most intensively studied as a FOXL2 target is follicle-stimulating hormone (Fshb) [119-121]. Pituitary and peripheral specialized Foxl2 deletions cause gonadotrope identification, even though basal and activin-stimulated FSH levels are much lower in both men and women. Male Foxl2 knockout mice have smaller testicles and spermatogenesis, whereas female Foxl2 knockout mice have lower ovarian weight and oogenesis. Recent findings [118] demonstrate that the main pituitary cells of Foxl2 mutant mice do not release FSH because of activin. Several studies have provided insight into the processes by which FOXL2 regulates Fshb expression. Together, FOXL2 and SMADs activate the Fshb genes in mice and pigs through activin [122-124]. Similarly, FOXL2 is involved in the Fshb promoter interaction between activins and progestins [125]. The impact of additional forkhead transcription factors on pituitary development is less well understood. Among the organs that contain FOXO1 are the ovary, brain, liver, pancreas, and adipose tissue [103, 126]. FOXO1 may have a function in cell cycle arrest because it is found in quiescent pituitary developing cells [127]. The pituitary cells that express FOXO1 are unknown. While FOXO1 was only found in gonadotropes that had functional inhibition of Lhb expression [128], one research [127] found it in one-tenth of gonadotropes and half of somatotrope cells. Further investigation is required to ascertain FOXO1's function in pituitary development. Between 9.5 and 10.5, the oral ectoderm that forms Rathke's pouch expresses FOXE1, temporarily described and necessary for thyroid organogenesis [129, 130]. The Foxe1 gene is not necessary for normal pituitary growth, as demonstrated by the lack of pituitary abnormalities in mice missing the gene [129].

A rare autoimmune hypophysitis causes the pituitary gland to become inflamed and produce fewer hormones. Although the precise mechanisms are unknown, several forkhead genes impact the generation of pituitary hormones and the immune system. For example, although not expressed in the maturing pituitary gland, FOXP3 and FOXD1 have an impact on pituitary hormone synthesis [131, 132]. FOXP3 is required for the growth and operation of regulatory T-cells and its loss results in a serious autoimmune illness [133, 134]. FOXP3 is indirectly essential for gonadotrope function, as evidenced by the decreased expression of the gonadotropins Lhb, Fshb, and Cga in mice with an inactivating Foxp3 mutant (scurfy mice) [132]. Foxd1 expresses at e10.5 not only in the kidney but also in the mesenchyma around the pituitary. Mice lacking foxd1 die 24 hours after birth from renal insufficiency [135, 136], showing a marked decrease in Lhb expression. Additionally, these mice show that the sella turcica has not developed enough [131].

Thus, pituitary function is impacted by both FOXD1 and FOXB3. According to their research, forkhead factors are essential for pituitary growth and operation. There is still much to learn about the role of this class of factors in pituitary organogenesis and hormone production. The members of the forkhead family may share functions, like those of many other transcription factor families.

### 3.1 Pituitary Progenitors

Newborn rats may have distinct cell types that are hormone producers [137]. Due to hormones secreted by the hypothalamus and physiological needs, each population grows after birth as the gland matures [138-143]. Certain hormone-producing cells rejoin the cell cycle during the expansion of postnatal organs [144-146]. When tissue is injured, the developed pituitary gland can regenerate to some extent [147-149]. Prolactin cells either recover slowly or not following ablation, although growth hormone production regenerates more slowly [150]. Because of this, it is unclear to what degree regeneration is possible and what mechanisms support it. It is established that the pituitary responds to physiological demands by proliferating terminally differentiated cells, trans-differentiating differentiated cells (*e.g.,* converting somatotrophs to lactotrophs), and differentiating progenitors/stem cells. Adult organs with high turnover and regeneration capacities, such as the liver, gut, skin, and bone marrow, were the first to be demonstrated to contain stem cells [151].

Additionally, organs such as the heart and brain [152, 153], which are mostly composed of post-mitotic cells, have been shown to harbor stem cells. There are stem cells in each of these organs, and they have three basic properties:

• The potential to divide and recombine itself.

• After cell loss, differentiate and rebuild tissue.

• The pituitary gland has a very slow rate of cell turnover [150].

Furthermore, specialized cells can reenter the cell cycle, although most cells that produce hormones do not undergo division [ 42 ]. In recent years, a significant amount of information about stem cells in the anterior pituitary has been uncovered [ 154 ]. Scientists have located the specific environment and connected it to Rathke's pouch in both humans and animals [ 155 ]. Understanding the stages involved in regulating multipotent progenitors and the signaling pathways that guide them toward specific cellular fates will be one of the upcoming challenges.

Pituitary stem cells were initially identified in 1969 as chromophobes, hormone-negative cells [156]. Mature acidophils (somatotrophs and lactotrophs) and basophils were produced when chromophobes were inserted into the hypothalamus of hypophysectomized rats (thyrotrophs, gonadotrophs, and corticotrophs). A short while later, a procedure was created allowing chromophobes to be separated into acidophils and basophils *in vitro* [157]. Recent studies have revealed that chromophobes are stem cells or pituitary progenitors that respond to hormones involved in hypothalamic signaling [158]. A deeper understanding of pituitary stem cells, progenitors, and transit-amplifying cells is necessary, as is the control of advancement *via* these processes. The different approaches to finding pituitary stem cells are covered in this paper. Because of their lengthy cytoplasmic extensions, follicular-stellate cells are non-granular cells that resemble stars. The anterior pituitary gland's parenchymal tissue is where they are found. S100 cells form a functional network that is regulated by the paracrine system in conjunction with endocrine cells. Their gap junctions and long cytoplasmic processes allow for intercellular communication. They function as scavenger cells and exhibit phagocytic activity [159].

Pituitary stem cells may originate from a subset of follicular-stellate cells, whereas another fraction may be involved in niche development or maintenance. To distinguish the distinct follicular-stellate cell groups and carry out a more exhaustive analysis of their function, new markers are required. *In vitro*, colonization is a capability of progenitors and stem cells. This was initially demonstrated for the pituitary by Thomas's group [160]. The production of CFC was restricted to AMCA-positive cells, which make up about 3.7% of pituitary cells. Of these cells, only 12.3% were able to produce CFC, indicating possible heterogeneity within the folliculo-stellate cell population [160]. Both the adult pituitary's residual part of Rathke's cleft and the cells lining the subluminal zone express angiotensin-converting enzymes. Precursor cell sources and niches are thought to be comprised of these [161]. AMCA-positive cells in CFC were enriched by cells selected for angiotensin converting enzyme but not SCA1 [161]. Further, 3.3% of AMCA-positive, GH-negative cells may develop *in vivo* and express GH six weeks after implantation [61]. Based on the ability of a fraction of follicular-stellate cells to form colonies *in vitro* and develop *in vivo*, these investigations have revealed the progenitor potential of these cells. To conclude that CFCs cause pituitary differentiation and self-renewal, there must be evidence of both. Pituitary stem cells comprise the CFCs that have these antigens. Vankelecom and associates found adult pituitary cells that have characteristics of stem or progenitor cells using a variety of methods [158]. While one approach relies on forming clonal spheres, the other is predicated on the notion that stem cells may filter out dangerous substances like Hoechst dye efflux. Sorting the bone marrow cells treated with Hoescht 33342 produced a fast-reflux cell pool, including markers for multipotential hematopoietic stem cells [162]. Numerous organs, including the pituitary gland, have been found to have stem cells using this technique [158, 163]. Sixty percent of the cells in this pituitary side population display high levels of the stem cell marker SCA1, whereas the remaining forty percent express low marker levels. Two pieces of evidence point to the non-high SCA1 fraction being the source of pituitary progenitor cells. The transcription factors Prop1, Hesx1, Pax6, and Lhx4, present in the progenitors of Rathke's pouch, are also expressed by this later group of cells. More importantly, all anterior pituitary endocrine cell types can only be produced by spheres formed by non-high SCA1 cells [164].

The finding [161] that progenitors are restricted to the angiotensin-converting enzyme positive, SCA1 negative fraction is consistent with this. More work is needed to characterize the non-high SCA1 cells to find markers that set them apart from the diverse side population and to ascertain their ability to self-renew and be pluripotent. Toreprogram established cells to become pluripotent stem cells, a collection of transcription factors common to stem cells must be stimulated [165]. This information was revealed in a series of experiments that earned Yamanaka and colleagues a Nobel Prize. Two pluripotency factors, SOX2 and OCT4, have been studied in pituitary tissue stem cells [155, 166]. Markers of pituitary progenitors that resemble stem cells in many ways include SOX2, SOX9, and OCT4 [167]. In humans and rats, SOX2, an HMG box transcription factor that is a member of the SOXB1 subfamily, is essential for the upkeep of several stem cell populations, including those in the central nervous system [168]. Rathke's pouch and around 3% of adult pituitary cells have an expression of SOX2, which has been seen lining the cleft and distributed throughout the parenchyma throughout development. A member of the SOXE family, SOX9 is a stem cell marker found in the central nervous system, pancreas, and retina [169-172]. By promoting differentiation along specific pathways, SOXE family members affect the function of SOXB1 family members in diverse organs [173, 174].

While SOX9's expression in the embryo seems to occur later than SOX2, it is expressed in the mature rodent pituitary gland similarly to SOX2. A small population of hormone-negative, SOX2-positive, SOX9-negative progenitors in the adult pituitary gland may form pituitary spheres *in vitro* that can self-renew, generating secondary spheres and developing into folliculo-stellate cells and all five of the AP's endocrine cells [166]. Though most of the requirements for stem cells are met by these traits, self-renewal must become apparent after at least five passes according to the traditional definition. If cultivated in an environment more nearly resembling the niche and inductive elements established by the ventral diencephalon's organizing center, they are likely to go through several cycles of self-renewal. It is most likely determined by cell-to-cell contact inside the niche whether to divide to produce transit-amplifying cells or to retain stem cells. The development of regenerative therapeutics requires identifying these pathways and defining the conditions that allow stem cell survival. GFRa2 (glial cell line-derived neurotrophic factor receptor alpha 2) was shown to be a marker of pituitary stem cells by Alvarez's group [155]. In addition to a small subset of cells dispersed across the anterior pituitary parenchyma, GFRa2 is present in 0.9% of adult pituitary cleft cells. These cells display many markers associated with stem cells, such as OCT4, SOX,2, and PROP1, an early-acting transcription factor exclusive to the pituitary that is needed to sustain all human pituitary cell types [175].

The five pituitary lineages can be formed by slowly proliferating GFPRa2+ PROP1+ cells, developing *in vitro*, and producing secondary pituitary spheres. For the progenitors and supporting cells to be identified, more markers are required. Proliferating cells round the remains of Rathke's cleft during embryonic development. Potential pituitary stem cells are found in a marginal or niche zone known as a multilayer zone. In addition to having many unbound ribosomes and polysomes, marginal cells also lack granularity and have a poorly constructed endoplasmic reticulum [154]. The discovery that cells lining the pituitary fissure adjacent to the peripheral region express nestin gave rise to the theory that marginal cells are stem cells [176]. A genomic approach revealed that an adult population of pituitary stem cells might express nestin and generate all the different kinds of differentiated anterior pituitary cells [177]. Nonetheless, it seems that some cells in Rathke's capsule are identified by the transgenic nestin expression as not producing endogenous nestin [178].

Nevertheless, it has been demonstrated that single progenitors in pituisphere cultures can create every kind of anterior pituitary cell [167]. A pool of pituitary progenitors may originate and be partly maintained by PROP1. Humans with PROP1 mutations experience increasing hormone deficiencies, often manifest as reduced gonadotropin, TSH, growth hormone production, and growth insufficiency. Pituitary hormone levels steadily decrease in the absence of therapy, and all anterior pituitary hormones, including ACTH, may finally disappear [175]. There is evidence that this evolution contributes to the transition from proliferation to differentiation, even if it does not seem to occur in mice [179]. PROP1 expression is shared by several stem cell markers, such as GFRa2, OCT4, and SOX2 [155, 180, 181]. During fetal pituitary organogenesis, additionally, a zone dividing differentiating from proliferating cells expresses Prop1. This transitional area also exhibits cyclin E and Notch2 expression [44, 55]. Prop1 synthesis is stimulated by notch signaling, indicating a feed-forward loop [53, 55].

More research is needed to find out which cell lineages derive from Prop1-expressing progenitors and if pituispheres may be produced by Prop1-expressing cells. Sasai and associates demonstrated, in a magnificent endeavor, that it is feasible to manipulate embryonic stem cells to duplicate Rathke's pouch development and generate differentiated, functional corticotrophs [182]. Remarkably, the stress response of hypophysectomies animals was restored just by transplanting these produced corticotrophs into their kidney capsule. It was astonishing enough to restore the stress response in hypophysectomized animals after transplanting these produced corticotrophs into their kidney capsules.The realization that WNT, Notch, BMP, and FGF signaling control anterior pituitary development and the neural ectoderm itself drove this differentiation laid the groundwork for the following treatments. It would be very intriguing if methods could be created to consistently guide the evolution of other lineages. Notch, BMP, and FGF signaling were the foundation for the interventions that directed this differentiation.

### 3.2 Regulation of Cell Cycle Process

**Fig. (3):**
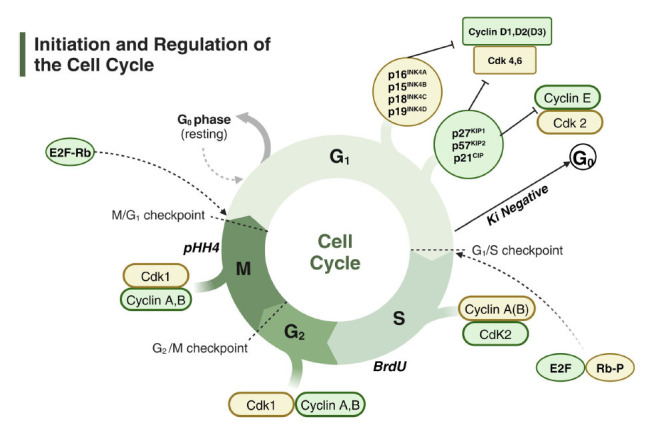
Initiation and regulation of the cell cycle.

It is crucial to regulate the transition from cell division to specialization, attract progenitor cells towards specialization, and prevent abnormal cell growth and the formation of adenomas to ensure proper organ development. A brief synopsis helps comprehend healthy pituitary development and pathological conditions since the cell cycle phases of eukaryotic cells, which range from yeast to humans, are essentially similar [183, 184]. During the synthetic (S) phase, the genetic makeup is duplicated, and during the mitotic (M) phase, it is split into two daughter cells. Gaps (G1 and G2) divide these two stages while the cell prepares for the following phase. The cell cycle transitioning from the G1 to the G0 phase typically coincides with cell differentiation (Fig. **3**).

The use of phospho-histone H3 (PHH3) immunostaining, complemented by bromodeoxyuridine (BrdU), is crucial in determining the synthesis (S) and mitosis (M) stages of the cell cycle. Quiescent cells, interestingly, do not show Ki67 staining. Rb phosphorylation, which breaks E2F's bond with Rb, signifies the G1/S phase transition. Various cyclins and cyclin-dependent kinases are expressed throughout the cell cycle, each playing a significant role.

Cell cycle regulation in humans and mice is an intricate process characterized by potential redundancy. This complexity is evidenced by over 30 cyclin genes and 25 cyclin-related genes. Cdkns work with their corresponding phase proteins to form protein complexes capable of halting cell cycle progression. The late G1 phase occurs when the tumor suppressor retinoblastoma protein, for example, becomes phosphorylated. This enables it to detach from the E2F1 transcription factor and activate genes that are necessary for the G1 to S phase transition. Rb stays phosphorylated and E2F1 is segregated during the M-phase, another crucial stage.

Global deletion of a single cell cycle regulator gene results in an atypical pituitary phenotype [185-196]. The most usually affected lobe is the intermediate lobe, which is primitive in humans and contains melanotropes in mice [77]. P57-/- mice display early-onset anterior pituitary hyperplasia, distinct from other knockout models that exhibit hyperplasia later. Additionally, the occurrence of adenomas at a high rate during late ages is a genetic background-dependent characteristic [197] and can make studying pituitary adenomas in mice more difficult. Pituitary gland hypoplasia is observed in Pttg1 and Cdk4 knockouts [198-200]. Because cell cycle regulators have overlapping activities, dual and triple loss of function mutations frequently result in more serious issues [44, 198, 201-204]. Cdks are critical regulators of the cell cycle. Knocking out Cdk2, Cdk4, and Cdk6 in mice leads to embryonic lethality around E14.5, indicating the essential role of Cdk1 in the cell cycle. Since the cell cycle is a vital process, there is a significant degree of functional redundancy and compensation among cell cycle regulators. In pituitary adenomas, researchers are examining cell cycle regulators, oncogenes, and tumor suppressors [205-207]. While there are some family variations of pituitary adenomas [18, 208], most pituitary adenomas are non-cancerous and arise spontaneously. Protein kinase mutations induce Carney Complex, whereas mutations in menin (MEN1) or cyclin-dependent kinase inhibitor 1B (p27, MEN4) cause multiple endocrine neoplasia, a genetic illness. Mutations in PRKAR1A result in an aryl hydrocarbon receptor interacting protein, regulatory subunit-1-alpha, and PRKAR1A. Current research explores the potential therapeutic advantages of drugs that modulate cell cycle regulators, specifically histone deacetylase inhibitors (HDACs). HDACs impede the activity of the p53 and p21 DNA-damage pathway [186, 209].

### 3.3 Hypophyseal Portal System and Vascularization

The hypophyseal portal system's development is essential to the pituitary gland's regular operation. The morphological development phases have been studied using a variety of techniques, including immunostaining, fluorescent gelatin, and India ink, but the molecular processes behind vascular expansion remain poorly known [15, 139, 210]. The direct correlation between vasculature invasion and pituitary differentiation activation remains uncertain. VEGF and FGF are two of the most well-researched angiogenic and anti-angiogenic factors expressed in the normal pituitary gland [211]. The timely production of VEGFA is crucial for vascularization in the pars distalis. This hormone-positive area, along with folliculostellate cells, exhibits VEGFA expression, resulting in higher vascularization in the pars distalis than in the pars intermedia. Ectopic production of VEGFA in the par’s intermedia leads to accelerated lobe growth but decreased expression of differentiation markers such as prohormone convertase two and MSH [212]. The abnormal growth of the portal system and hypophyseal arteries in some infants with hypopituitarism could be an outcome, not a trigger, of the disorder [213]. In animals with multiple endocrine neoplasia, however, research indicates that anti-VEGFA antibody treatment can lower blood prolactin levels and pituitary growth. This suggests that vascularization may affect the differentiation of the pars distalis in the pituitary gland [214]. Prop1 mutant pituitaries produce VEGFA, but their vascularization is inadequate, their differentiation is unsuccessful, and their apoptosis rate is elevated [139]. These findings suggest that VEGFA expression is not enough for normal angiogenesis. The mechanisms governing appropriate vascularization and how vascularization influences pituitary differentiation require more investigation.

## CONCLUSION

We can better understand pituitary development by using different models and technologies. We can track changes in chromatin accessibility and gene expression during development and in specific cells using next-generation sequencing and analytics. Zebrafish can stimulate or inhibit gene expression during embryonic development, while frogs and chicks can have tissue transplants at any point in their growth. Scientists have recently made advances in modifying stem cells to produce hormones in mice. Finally, relevant genes can always be identified in human patients.

## Figures and Tables

**Table 1:** Pituitary transcription factor in the pituitary function aspect of human illness.

**Human Disease**	**Pituitary Function**	**References**
Polydactyly, Congenital club foot, Liebenberg syndrome (*i.e.,* homeotic arm to leg transformation)	Modest, overlaps with PITX2	[46]
Rieger Syndrome, Eyes, teeth, umbilicus	Rathke’s pouch expansion	[47]
Septo-optic-dysplasia, moderate to severe hypopituitarism	Affects pituitary growth and midline	[48]
Evolving hypopituitarism	OTX2, Silencing HESX1, and Activating POU1F1, NOTCH2	[49]
Different optic and eye nerve anomalies	Expansion of TSH hormone cells increased growth at the expense of GH and LH	[50]
Alveolar, Rhabdomyosarcoma-2	Remodeling of Chromatin for the selection of melanotrope fate	[51]

## LIST OF ABBREVIATIONS

ACTH: Adrenocorticotropic Hormone
BEPS: Blepharophimosis, and Epicanthus Inversus Syndrome
GH: Growth Hormone
PRL: Prolactin
Shh: Sonic Hedgehog
